# Supplementary material for: An Overview of the Evolution of Capsule Endoscopy Research—Text-Mining Analysis and Publication Trends
Source: Diagnostics (Basel). 2022 Sep 16;12(9):2238. doi: 10.3390/diagnostics12092238 (PMC9498258; doi:10.3390/diagnostics12092238)

Title: An Overview of the Evolution of Capsule Endoscopy Research—Text-Mining Analysis and Publication Trends.

Supplementary Material

Figure S1. Capsule endoscopy publications included in the study, according to research areas.

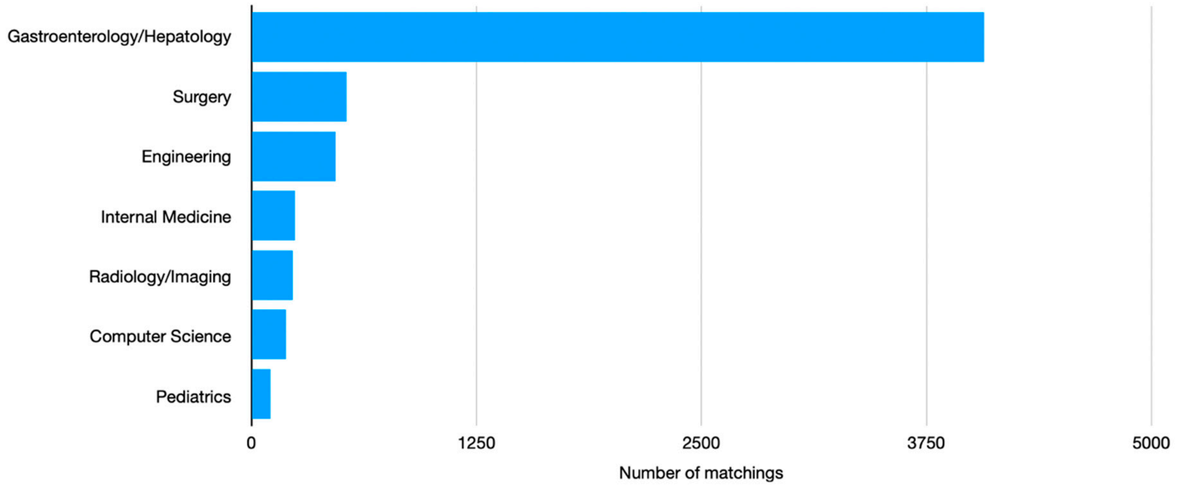

Table S1: Top 10 capsule endoscopy publication sources, 2000 to 2021.

Source	Publications	Impact Factor	% of 5,734
Gastrointestinal Endoscopy	997	9.427	32.8
American Journal of Gastroenterology	469	10.864	15.4
Gastroenterology	328	22.682	10.8
Journal of Gastroenterology and Hepatology	247	4.029	8.1
Endoscopy	191	10.093	6.3
Gut	190	23.059	6.3
World Journal of Gastroenterology	170	5.742	5.6
Digestive and Liver Disease	166	4.088	5.5
Digestive Diseases and Sciences	127	3.199	4.2
European Journal of Gastroenterology and Hepatology	77	2.566	2.5
Scandinavian Journal of Gastroenterology	77	2.423	2.5

Table S2: 10 most cited capsule endoscopy articles

Authors	Year	Title/DOI	Source	TC
Iddan G. et al.	2000	*Wireless Capsule Endoscopy* https://doi.org/10.1038/35013140	Nature	1954

Van Assche G. et al.	2010	*The Second European Evidence-based Consensus on the Diagnosis and Management of Crohn's Disease: Definitions and Diagnosis* https://doi.org/10.1016/j.crohns.2009.12.003	J Crohns Colitis	796
Pennazio M. et al.	2004	*Outcome of Patients With Obscure Gastrointestinal Bleeding After Capsule Endoscopy: Report of 100 Consecutive Cases* https://doi.org/10.1053/j.gastro.2003.11.057	Gastroenterology	678
Costamagna G. et al.	2002	*A Prospective Trial Comparing Small Bowel Radiographs and Video Capsule Endoscopy for Suspected Small Bowel Disease* https://doi.org/10.1053/gast.2002.35988	Gastroenterology	625
Levine A. et al.	2014	*ESPGHAN Revised Porto Criteria for the Diagnosis of Inflammatory Bowel Disease in Children and Adolescents* 10.1097/MPG.0000000000000239	J Pediatr Gastroenterol Nutr	621
Van Assche G. et al.	2010	*The Second European Evidence-based Consensus on the Diagnosis and Management of Crohn's Disease: Special Situations* https://doi.org/10.1016/j.crohns.2009.09.009	J Crohns Colitis	540
Ell C. et al.	2002	*The First Prospective Controlled Trial Comparing Wireless Capsule Endoscopy with Push Enteroscopy in Chronic Gastrointestinal Bleeding* DOI: 10.1055/s-2002-33446	Endoscopy	515
Annese V. et al.	2013	*European Evidence Based Consensus for Endoscopy in Inflammatory Bowel Disease* https://doi.org/10.1016/j.crohns.2013.09.016	J Crohns Colitis	488
Yamamoto H. et al.	2004	*Clinical Outcomes of Double-Balloon Endoscopy for the Diagnosis and Treatment of Small-Intestinal Diseases* https://doi.org/10.1016/S1542-3565(04)00453-7	Clin Gastroenterol Hepatol	484
Goldstein J. et al.	2005	*Video Capsule Endoscopy to Prospectively Assess Small Bowel Injury With Celecoxib, Naproxen Plus Omeprazole, and Placebo* https://doi.org/10.1016/S1542-3565(04)00619-6	Clin Gastroenterol Hepatol	479

***Note**: TC = Total Citations, TCperYear = Total Citations per Year*

Figure S2: grouped display of capsule endoscopy publications per keywords, in different timeframes

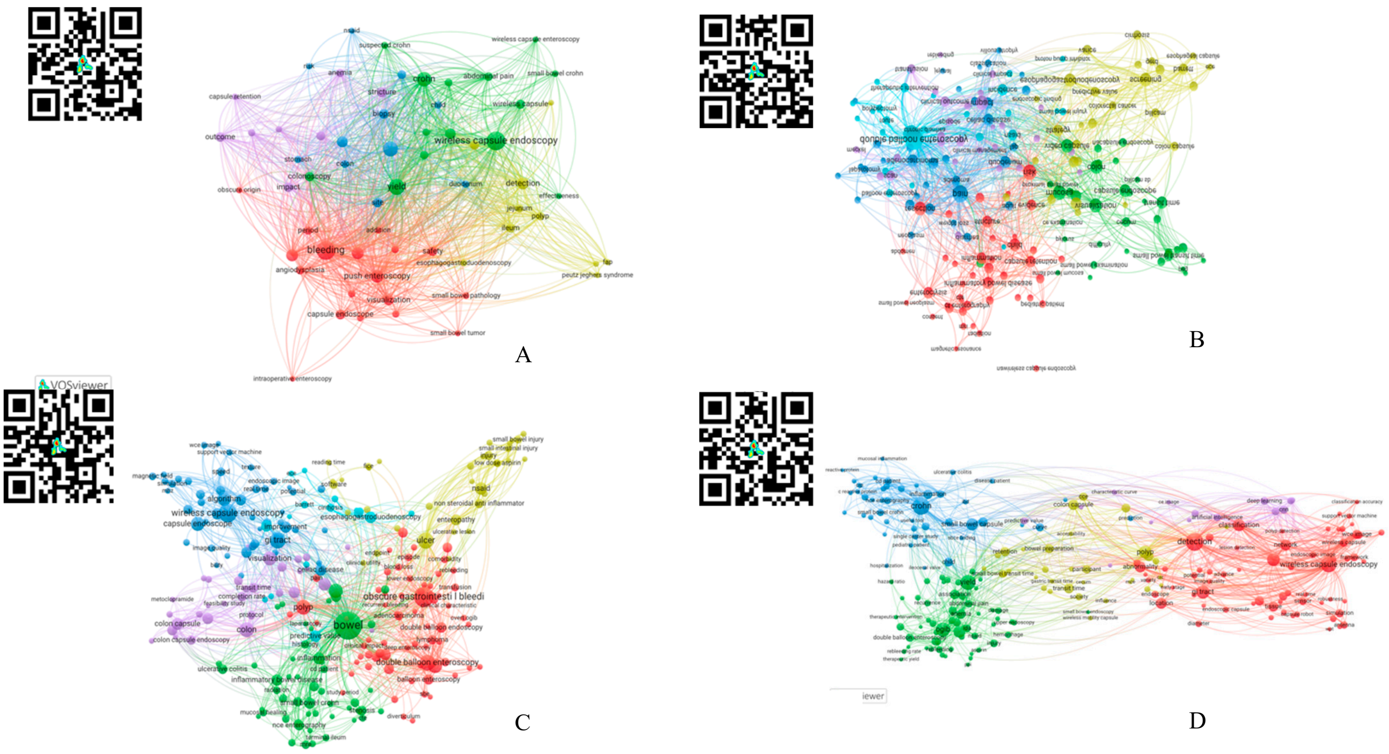

A: Time segment 1 *– 2000-2005; 5,372 terms, min. of 10 occurrences, 60% most relevant terms = 61 terms; https://tinyurl.com/y7wg76oz*
B: Time segment 2 *– 2006-2010; 16,671 terms; min. of 10 occurrences, 60% most relevant terms = 193; https://tinyurl.com/y9hhfw7d*
C: Time segment 3 *– 2011-2016; 23,070 terms; min 10 occurrences, 60% most relevant terms = 250; https://tinyurl.com/y7z3vp4m*
D: Time segment 4 *– 2017-2021; 23,429 terms; min 10 occurrences, 60% most relevant terms = 215; https://tinyurl.com/ybecgs3q*

Table S3: capsule endoscopy publications per capsule type, 2000 to 2021

Type	Documents	Top 3 contributing countries
CCE	n = 349	Japan (n = 53; TC = 273)<br>Italy (n = 39; TC = 1283)<br>USA (n = 19; TC =740)
ECE	n = 127	USA (n = 40; TC = 917)<br>France (n = 11; TC = 287)<br>Italy (n = 8; TC = 179)
MCE	n = 46	China (n = 14; TC = 60)<br>USA (n = 9; TC = 693)<br>Italy (n = 5; TC =83)
NIC	n = 215	USA (n = 70; TC = 1,786)<br>Denmark (n = 13; TC = 101)<br>United Kingdom (n = 12; TC = 264)
RC	n = 73	China (n = 43; TC = 397)<br>USA (n = 31 TC = 698)<br>Australia (n = 10; TC = 163)

SBCE	n = 686	China (n = 96; TC = 1,749)<br>USA (n = ; TC = 2,008)<br>Japan (n = 54; TC =444)

Note: *Contributing countries by country of origin of corresponding authors;*
RC, Robotic Capsule; MCE, Magnetic Capsule; CCE, Colon Capsule; ECE, Esophageal Capsule; NIC, Non-imaging Capsule; SBCE, Small-Bowel Capsule.

Table S4: number of capsule endoscopy publications per year, 2000 to 2021

Year	Number of Documents
2000	8
2001	8
2002	74
2003	104
2004	188
2005	264
2006	321
2007	311
2008	339
2009	330
2010	365
2011	308
2012	353
2013	286
2014	288
2015	336
2016	308
2017	297
2018	306
2019	329
2020	290
2021	321

Per country

**Country of Origin (first author)**	**Number of Documents**
NA	*1385*
USA	1048
Japan	592
China	488
Italy	309
United Kingdom	234

Korea	178
Germany	176
France	151
Spain	117
Israel	112
Portugal	109
Canada	89
Australia	79
Netherlands	74
Greece	66
India	66
Belgium	47
Turkey	42
Denmark	29
Sweden	29
Ireland	27
Romania	26
Singapore	25
Czech Republic	19
Poland	19
Norway	16
Iran	14
Thailand	14
Finland	12
Pakistan	12
Switzerland	12
Brazil	11
Malaysia	10
Morocco	10
Austria	9
Bangladesh	8
Egypt	8
Hungary	7
Mexiko	7
Saudi Arabia	6
New Zealand	5
Serbia	5
Russia	3
South Afrika	3
Vietnam	3
Algeria	2
Colombia	2
Philippines	2

Slovakia	2
Slovenia	2
Angola	1
Argentina	1
Bahamas	1
Bosnia	1
Chile	1
Cyprus	1
Ecuador	1
Georgia	1
Indonesia	1
Jordan	1
Kuwait	1
Lebanon	1
Mali	1

Table S5: contribution in capsule endoscopy publications by corresponding authors' countries of origin

Country	Docs.	TC	Norm.C.	Avg. pub. Year	Avg. C.	Avg. norm. C.
Algeria	5	105	18.2	2019.2	21	3.6
Argentina	9	657	48.2	2014.3	73	5.4
Australia	147	1702	94.2	2012.4	11.6	0.6
Austria	15	1102	75.7	2013.7	73.5	5.0
Bangladesh	9	198	19.2	2017	22	2.1
Belgium	71	4836	314.6	2013.1	68.1	4.4
Brazil	18	509	62.2	2013.9	28.3	3.5
Canada	138	4194	340.6	2014.3	30.4	2.5
Cyprus	6	78	13.2	2017.7	13	2.2
Czech Republic	27	1129	65.8	2013.6	41.8	2.4
Denmark	38	1858	156.8	2016.1	48.9	4.1
Egypt	15	218	22.1	2015.3	14.5	1.5
Finland	21	1473	86.1	2013.1	70.1	4.1
France	177	8490	501.3	2013.1	47.9	2.8
Germany	229	11427	640.0	2011.3	49.9	2.8
Greece	81	4976	294.6	2012.5	61.4	3.6
Hungary	11	725	39.3	2011.8	65.9	3.6
India	79	941	97.8	2015.5	11.9	1.2
Iran	20	103	14.9	2017.9	5.2	0.7
Ireland	43	751	70.3	2015.9	17.5	1.6
Israel	173	9920	492.7	2011.9	57.3	2.8
Italy	437	14698	827.4	2012.7	33.6	1.9

Japan	585	8591	555.1	2014.4	14.7	0.9
Lebanon	6	60	13.9	2020	10	2.3
Malaysia	16	364	35.8	2017.1	22.8	2.2
Malta	8	462	68.5	2019.6	57.8	8.6
Mexico	16	252	21.2	2015.7	15.8	1.3
Morocco	13	109	14.7	2017.5	8.4	1.1
Netherlands	98	5467	351.1	2012	55.8	3.6
New Zealand	15	355	20.8	2015	23.7	1.4
Norway	32	670	55.1	2015.1	20.9	1.7
Pakistan	24	495	80.9	2018.64	20.6	3.4
China	436	6519	512.2	2015.8	14.9	1.2
Poland	31	1107	90.3	2016.8	35.7	2.9
Portugal	137	4454	316.6	2016.4	32.5	2.3
Romania	31	353	22.6	2013.6	11.4	0.7
Saudi Arabia	15	257	45.7	2017.6	17.1	3.0
Serbia	8	66	4.6	2013.5	8.3	0.6
Singapore	32	562	42.9	2013.5	17.6	1.3
South Africa	5	42	6.4	2014.8	8.4	1.3
South Korea	186	3428	268.4	2014.4	18.4	1.4
Spain	171	6477	425.9	2013.3	37.9	2.5
Sweden	57	1918	122.1	2012.9	33.7	2.1
Switzerland	33	2672	173.4	2012.3	80.9	5.3
Taiwan	45	661	45.3	2012.2	14.7	1.0
Thailand	16	76	6.7	2015.6	4.8	0.4
Turkey	41	358	24.7	2012.5	8.7	0.6
United Arab. Emirates	5	503	67.8	2018.8	100.6	13.6
United Kingdom	461	12941	783.8	2013.8	28.1	1.7
USA	1229	26622	1534.0	2011.9	21.7	1.2

Note: *Docs = Documents, TC = Total Citations, Norm.C. = Normalized Citations, Avg. pub. Year = Average publication year, Avg. C.= Average Citations, Avg. norm. C. = Average normalized Citations*

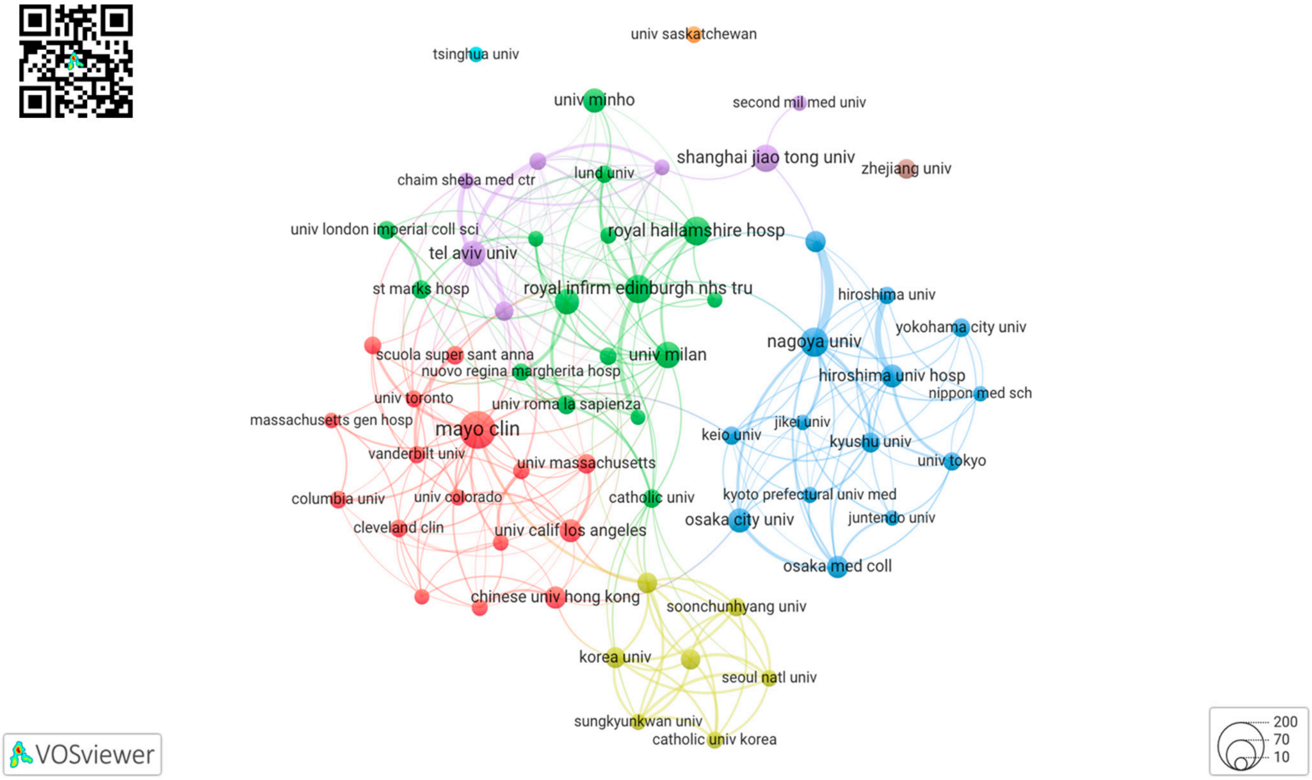

Figure S3: authorship collaboration co-occurrence network
An interactive visualization is available at https://tinyurl.com/yc6vtgvh or via the QR-code above.
Layout settings in VOSviewer: attraction = 2, repulsion = -2 , resolution = 1, min. cluster size = 1

Organization	Cluster	Docs	TC	Norm. C	Avg. pub. Year	Avg.C	Avg. norm.C
Mayo Clinic, USA	1	117	4920	243.3	2012.1	42.1	2.1
Nagoya University, Japan	3	70	1235	105.3	2015.4	17.6	1.5
Royal Hallamshire Hospital, UK	2	67	741	45.6	2012.8	11.1	0.7
Royal Infirmary of Edinburgh, UK	2	66	797	78.1	2015.3	12.1	1.2
Shanghai Jiao Tong University, China	5	62	1170	71.4	2014.4	18.9	1.2
University Milan, Italy	2	58	2745	128.3	2012.3	47.3	2.2
Tel Aviv University, Israel	5	55	1775	133.7	2014.2	32.3	2.4
Università Cattolica del Sacro Cuore, Italy	2	53	2171	142.0	2013.4	41.0	2.7
Osaka City University, Japan	3	48	905	57.7	2014.2	18.9	1.2
University of Minho, Portugal	2	48	354	38.7	2018	7.4	0.8
Hiroshima University Hospital, Japan	3	44	792	73.7	2015.6	18.0	1.7
University of California, USA	1	44	440	31.9	2012.9	10.0	0.7
The Chinese University of Hong Kong, China	1	41	1518	110.6	2013.1	37.0	2.7
Osaka Medical College, Japan	3	41	665	41.2	2015.4	16.2	1.0
Nagoya University Hospital, Japan	3	37	603	39.5	2015.3	16.3	1.1

Korea University, Korea	4	36	608	43.5	2013.4	16.9	1.2
University of Ulsan, Korea	4	35	968	59.2	2013.7	27.7	1.7
Yonsei University, Korea	4	34	791	47.3	2012.6	23.3	1.4
Kyushu University, Japan	3	33	899	50.8	2013.3	27.2	1.5
University of Massachusetts, USA	1	32	347	26.0	2014.5	10.8	0.8
Zhejiang University of Science, China	8	32	504	34.4	2014.9	15.8	1.1
Technion - Israel Institute of Technology	5	31	1609	62.6	2010.3	51.9	2.0
University Roma la Sapienza, Italy	2	30	1760	104.3	2012.6	58.7	3.5
Catholic University, Rome, Italy	2	29	1393	71.2	2011.8	48.0	2.5
Scuola Superiore Sant'Anna di Pisa, Italy	1	29	1385	71.8	2012.6	47.8	2.5
Soonchunhyang University, Korea	4	29	274	34.4	2017.4	9.4	1.2
St Mark's Hospital, London/UK	2	29	623	54.3	2011.2	21.5	1.9
Yokohama City University, Japan	3	29	505	29.9	2013.1	17.4	1.0
Keio University, Korea	3	28	439	41.2	2014.1	15.7	1.5
Imperial College of Science, Technology and Medicine, London	2	28	1053	58.9	2010.9	37.6	2.1
University of Tokyo, Japan	3	28	474	55.7	2015.3	16.9	2.0
Cleveland Clinic, USA	1	26	484	53.8	2011.8	18.6	2.1
Columbia Unversity, USA	1	26	712	59.7	2013.8	27.4	2.3
Indiana University Bloomington, USA	1	26	782	50.6	2010.6	30.1	1.9
Lund University, Sweden	2	26	259	32.4	2014.3	10.0	1.2
Hiroshima University, Japan	3	25	711	39.9	2013.7	28.4	1.6
Ospedale Nuovo Regina Margherita, Italy	2	25	673	69.2	2016.6	26.9	2.8
Sheba Medical Center, Israel	5	25	739	69.7	2017.7	29.6	2.8
Vanderbilt University, USA	1	25	1016	77.2	2014.1	40.6	3.1
Catholic University of Korea, Korea	4	24	432	32.5	2013.6	18.0	1.4
Oregon Health & Science University, USA	1	24	1017	55.7	2011.2	42.4	2.3
University of Toronto, Canada	1	24	1670	146.4	2013	69.6	6.1
Seoul National University, Korea	4	23	588	39.0	2013.1	25.6	1.7
Stanford University, USA	1	23	1114	50.7	2010.9	48.4	2.2
University of Athens, Greece	2	23	686	33.3	2010.1	29.8	1.4
University of Saskatchewan, Canada	7	23	328	26.1	2016.4	14.3	1.1
Sheba Medical Center, Israel	5	22	484	31.0	2012.8	22.0	1.4
Kyoto Prefectural University of Medicine, Japan	3	22	327	21.3	2013.5	14.9	1.0
Sungkyunkwan University, Korea	4	22	487	32.1	2013	22.1	1.5
University of Colorado Boulder, USA	1	22	404	49.6	2016.4	18.4	2.3
University of Michigan, USA	1	22	442	25.4	2012.6	20.1	1.2
Hôpital Edouard Herriot, France	2	21	1236	71.6	2013	58.9	3.4
McGill University, Japan	5	21	275	21.5	2014.6	13.1	1.0
Sheffield Teaching Hospital, UK	2	21	39	5.6	2017.5	1.9	0.3
Jikei University School of Medicine, Japan	3	20	226	27.3	2015.7	11.3	1.4

Juntendo University, Japan	3	20	130	19.4	2015.9	6.5	1.0
Massachusetts General Hospital, USA	1	20	705	61.8	2015.9	35.3	3.1
Nippon Medical School, Japan	3	20	440	23.3	2013.9	22.0	1.2
Second Military Medical University, Chihna	5	20	787	51.4	2015.2	39.4	2.6
Tsinghua University, China	6	20	214	19.2	2014.6	10.7	1.0
University of Edinburgh, UK	2	20	876	54.5	2015.5	43.8	2.7
Washington University, USA	1	20	434	28.2	2012.7	21.7	1.4

Note: Docs = Documents, TC = Total Citations, Norm.C. = Normalized Citations, Avg.pub.Year = Average publication year, Avg.C.= Average Citations, Avg.norm.C. = Average normalized Citations

Key terms

Keyword	Occurrences	Avg.pub.Year	Avg.Citations	Avg.norm. Citations
Diagnostic Yield	1126	2012.8	26.9	1.5
IBD	629	2012.3	34.0	1.9
OGIB	580	2012.7	23.0	1.2
Cancer	484	2012.9	25.9	1.5
Disease	335	2011.6	28.3	1.5
Lesions	242	2013.2	22.7	1.3
Clinical Impact	235	2012.7	31.2	1.7
Experience	203	2010.9	34.0	1.5
Imaging	202	2014.0	20.5	1.6
Risk Factors	200	2014.1	22.6	1.4
Polyposis	160	2012.5	29.7	1.8
NSAID	157	2013.3	29.9	1.7
Injury	156	2013.6	26.1	1.5
ICT	142	2012.9	29.5	1.6
Celiac Disease	139	2012.7	27.1	1.7
Complications	139	2012.6	24.4	1.3
Transit time	134	2012.8	21.9	1.3
Artificial Intelligence	130	2017.9	19.9	2.6
Pediatric patients	126	2013.0	19.1	1.1
Retention	122	2013.8	20.1	1.3
IDA	105	2013.9	19.3	1.3

Polyps	100	2013.5	20.3	1.4
Classification	95	2016.0	33.8	2.7
Bowel preparation	84	2013.2	25.9	1.6
Angiodysplasia	83	2013.4	21.8	1.2
Biomarker	80	2016.3	43.2	3.3
Surveillance	80	2012.6	29.5	1.7
Portal hypertension	79	2012.6	16.2	0.9
Outcome	73	2015.0	23.7	1.7
PPI	67	2012.9	23.4	1.3
Barrett's esophagus	63	2013.5	21.4	1.5
Patency	62	2012.9	38.8	2.2
GERD	60	2010.7	22.2	1.1
Pathology	58	2012.1	35.8	2.1
Validation	58	2016.0	23.5	2.0
Activity index	54	2015.6	20.6	1.6
Cirrhosis	53	2012.8	15.9	0.9
Safety	50	2013.3	23.8	1.4
Motility	49	2013.9	17.1	1.1
Ulcer	47	2011.0	26.8	1.5
Recurrence	46	2013.5	31.7	1.7
Prevention	45	2013.0	25.6	1.7
Helicobacter pylori	43	2012.2	19.8	1.2
Prokinetics	41	2012.6	20.4	1.0
Obstruction	40	2011.6	22.2	1.2
Stenosis	40	2013.0	19.5	1.1
Resection	39	2013.2	52.2	4.0
Screening	38	2013.3	19.7	1.4
Abdominal pain	36	2011.8	26.5	1.6
Biopsy	36	2013.5	25.1	1.6
Atrophy	35	2013.3	26.5	1.9
Localization	35	2015.2	14.6	1.3
Completion rate	34	2015.7	13.1	1.0
Simethicone	34	2015.2	17.5	1.4
Mortality	31	2014.0	24.0	1.6
Cost effectiveness	30	2013.3	18.7	1.4
Enteritis	30	2011.3	39.4	1.6
Prognosis	30	2012.5	23.0	1.3
Rheumatoid arthritis	28	2012.3	38.2	2.0
Gluten free diet	27	2013.6	31.6	1.7
Adult patients	26	2013.8	17.8	1.1
Infliximab	25	2014.0	39.9	2.3
Robotics	25	2016.4	40.0	3.0
Antennas	24	2018.5	12.9	1.4
Locomotion	21	2016.8	26.0	2.1
Intussusception	20	2012.4	19.2	1.0
Transmission	20	2014.6	24.5	1.7
Meckel's diverticulum	19	2011.4	14.1	0.6

Prediction	19	2015.1	18.9	1.4
Insertion	18	2009.8	76.8	2.9
Permeability	18	2011.7	58.4	2.5
Vascular malformations	18	2012.4	32.1	1.8
Diarrhea	17	2013.6	13.7	0.8
Expression	16	2010.9	16.2	0.8
Infection	16	2012.2	14.1	0.6
Score	16	2017.5	15.1	1.6
Survival	16	2012.4	25.4	1.5
Angiodisplasia	15	2010.9	16.5	0.8
Colitis	15	2012.4	47.2	2.3
Esophageal	15	2012.1	21.5	1.6
Jejunum	15	2013.0	11.1	0.6
Recognition	15	2018.7	27.5	4.3
Reconstruction	15	2014.9	36.5	2.7
Small-bowel diseases	15	2010.4	31.5	1.7

Note: *Docs = Documents, TC = Total Citations, Norm.C. = Normalized Citations, Avg. pub. Year = Average publication year, Avg. C.= Average Citations, Avg. norm. C. = Average normalized Citations*

Most cited publications per capsule type

1. CCE

Author(s)	Year	Source	Title	TC	TCper Year	NTC
VAN GOSSUM, A. et al.	2009	N ENGL J MED	*Capsule Endoscopy versus Colonoscopy for the Detection of Polyps and Cancer* DOI: 10.1056/NEJMoa0806347	252	18	4.5
ELIAKIM, R. et al.	2006	ENDOSCOPY	*Evaluation of the PillCam Colon capsule in the detection of colonic pathology: results of the first multicenter, prospective, comparative study* DOI: 10.1055/s-2006-944832	251	14.76	1.138
ELIAKIM,R . et al.	2009	ENDOSCOPY	*Prospective multicenter performance evaluation of the second-generation colon capsule compared with colonoscopy* DOI: 10.1055/s-0029-1215360	208	14.86	3.714
SCHOOFS, N., DEVIERE, J. & VAN GOSSUM, A.	2006	ENDOSCOPY	*PillCam colon capsule endoscopy compared with colonoscopy for colorectal tumor diagnosis: a prospective pilot study* DOI: 10.1055/s-2006-944835	190	11.18	0.862
SPADA, C. et al.	2011	GASTROINTES T ENDOSC	*Second-generation colon capsule endoscopy compared with colonoscopy* DOI: 10.1016/j.gie.2011.03.1125	186	15.5	7.417

2. SBCE

Author(s)	Year	Source	Title	TC	TCpe r	NTC

					Year	
PENNAZIO, M. et al.	2015	ENDOSCOPY	*Small-bowel capsule endoscopy and device-assisted enteroscopy for diagnosis and treatment of small-bowel disorders: European Society of Gastrointestinal Endoscopy (ESGE) Clinical Guideline* DOI: 10.1055/s-0034-1391855	412	51.5	14.91
LIAO, Z. et al.	2010	GASTROINTEST ENDOSC	*Indications and detection, completion, and retention rates of small-bowel capsule endoscopy: a systematic review* DOI: 10.1016/j.gie.2009.09.031	407	31.31	13.451
ENNS, R.A. et al.	2017	GASTROENTEROL-OGY	*Clinical Practice Guidelines for the Use of Video Capsule Endoscopy* DOI: 10.1053/j.gastro.2016.12.032	170	28.33	12.897
DELVAUX, M., FASSLER, I. & GAY, G.	2004	ENDOSCOPY	Clinical Usefulness of the Endoscopic Video Capsule as the Initial Intestinal Investigation in Patients with Obscure Digestive Bleeding: Validation of a Diagnostic Strategy Based on the Patient Outcome after 12 Months DOI: 10.1055/s-2004-826034	157	8.26	1.019
ADLER, D.G., KNIPSCHIELD, M. & GOSTOUT, Ch.	2004	GASTROINTEST ENDOSC	A prospective comparison of capsule endoscopy and push enteroscopy in patients with GI bleeding of obscure origin DOI: 10.1016/S0016-5107(03)02862-1	151	7.95	0.981

3. RC

Author(s)	Year	Source	Title	TC	TCper Year	NTC
MOGLIA, A. et al.	2007	BIOMED MICRODEVICES	*Wireless capsule endoscopy: from diagnostic devices to multipurpose robotic systems* https://doi.org/10.1007/s10544-006-9025-3	159	9.94	1
VALDASTRI, P. et al.	2009	IEEE TRANS ROBOT	*A New Mechanism for Mesoscale Legged Locomotion in Compliant Tubular Environments* DOI:10.1109/TRO.2009.2014127	158	11.29	1
GLASS, P., CHEUNG; E. & SITTI; M.	2008	IEEE TRANS BIOMED ENG	*A Legged Anchoring Mechanism for Capsule Endoscopes Using Micropatterned Adhesives* DOI:10.1109/TBME.2008.2002111	118	7.87	1
YIM, S. & SITTI, M.	2012	IEEE TRANS ROBOT	*Shape-Programmable Soft Capsule Robots for Semi-Implantable Drug Delivery* DOI: 10.1109/TRO.2012.2197309	77	7	1.593

VALDASTRI, P. et al.	2012	SURG ENDOSC	*Magnetic air capsule robotic system: proof of concept of a novel approach for painless colonoscopy* DOI: 10.1007/s00464-011-2054-x	56	5.09	1.159

4. *ECE*

Author(s)	Year	Source	Title	TC	TCper Year	NTC
EISEN, G.M. et al.	2006	BIOMED MICRODEVICES	*The Accuracy of PillCam ESO Capsule Endoscopy Versus Conventional Upper Endoscopy for the Diagnosis of Esophageal Varices: A Prospective Three-Center Pilot Study* DOI: 10.1055/s-2005-921189	120	7.06	2.94
DE FRANCHIS, R. et al.	2008	HEPATOLOGY	*Esophageal capsule endoscopy for screening and surveillance of esophageal varices in patients with portal hypertension* https://doi.org/10.1002/hep.22227	110	7.33	4.76
ELIAKIM, R. et al.	2005	J CLIN GASTROENTEROL	*A Prospective Study of the Diagnostic Accuracy of PillCam ESO Esophageal Capsule Endoscopy Versus Conventional Upper Endoscopy in Patients With Chronic Gastroesophageal Reflux Diseases* DOI: 10.1097/01.mcg.0000170764.29202.24	104	5.78	3.8
ELIAKIM, R., SHLOMI, I. & EISEN, G. M.	2004	ALIMENT PHARMACOL THER	*A novel diagnostic tool for detecting oesophageal pathology: the PillCam oesophageal video capsule* https://doi.org/10.1111/j.1365-2036.2004.02206.x	100	5.26	2.91
REY, J.F. et al.	2006	ENDOSCOPY	*European Society of Gastrointestinal Endoscopy (ESGE) Video capsule endoscopy: Update to guidelines (May 2006)* DOI: 10.1055/s-2006-944874	98	5.76	2.4

5. *MCE*

Author(s)	Year	Source	Title	TC	TCper Year	NTC
YIM, S. & SITTI, M.	2012	IEEE TRANS ROBOT	*Design and Rolling Locomotion of a Magnetically Actuated Soft Capsule Endoscope* DOI: 10.1109/TRO.2011.2163861	200	18.18	1.444
YIM, S. et al.	2014	IEEE TRANS BIOMED ENG	*Biopsy using a Magnetic Capsule Endoscope Carrying, Releasing, and Retrieving Untethered Microgrippers* DOI: 10.1109/TBME.2013.2283369	134	14.89	1
MAHONEY, A.W. & ABBOT, J. J.	2016	INT J ROBOT RES	*Five-degree-of-freedom manipulation of an untethered magnetic device in fluid using a single permanent magnet with application in stomach capsule endoscopy* https://doi.org/10.1177/0278364914558006	97	13.86	1.805
LIAO, Z. et al.	2016	CLIN GASTROENTEROL HEPATOL	*Accuracy of Magnetically Controlled Capsule Endoscopy, Compared With Conventional Gastroscopy, in Detection of Gastric Diseases*	92	13.14	1.712

			https://doi.org/10.1016/j.cgh.2016.05.013			
YIM, S. & SITTI, M.	2012	IEEE TRANS ROBOT-a	*Shape-Programmable Soft Capsule Robots for Semi-Implantable Drug Delivery* DOI: 10.1109/TRO.2012.2197309	77	7	0.556

6. *NIC*

**Author(s)**	**Year**	**Source**	**Title**	**TC**	**TCper Year**	**NTC**
RAO,S.S.C. et al.	2011	NEUROGASTRO-ENTEROL MOTIL	*Evaluation of gastrointestinal transit in clinical practice: position paper of the American and European Neurogastroenterology and Motility Societies* https://doi.org/10.1111/j.1365-2982.2010.01612.x	216	18	8.16
RAO, S.S.C. et al.	2009	CLIN GASTROENTEROL HEPATOL	*Investigation of Colonic and Whole-Gut Transit With Wireless Motility Capsule and Radiopaque Markers in Constipation* https://doi.org/10.1016/j.cgh.2009.01.017	205	14.64	2.253
SAAD, R.J. et al.	2010	AM J GASTROENTEROL	*Do Stool Form and Frequency Correlate With Whole-Gut and Colonic Transit? Results From a Multicenter Study in Constipated Individuals and Healthy Control* 10.1038/ajg.2009.612	148	11.38	4.388
RAO, S.S.C., RATTANA-KOVIT, K. & PATCHARA-TRAKUL, T.	2016	NAT REV GASTROENTEROL HEPATOL	*Diagnosis and management of chronic constipation in adults* https://doi.org/10.1038/nrgastro.2016.53	116	16.57	10.157
CAMILLERI, M. et al.	2010	NEUROGASTROENTEROL MOTIL	*Wireless pH-motility capsule for colonic transit: prospective comparison with radiopaque markers in chronic constipation* https://doi.org/10.1111/j.1365-2982.2010.01517.x	106	8.15	3.143

Figure S4: appendix keyterms co-occurrence map

Layout settings VosViewer: Attraction = 1; Repulsion =- 3; Resolution = 1; min. Cluster size =1; an online visualization of the clustered terms is available at https://tinyurl.com/y83ulbdo

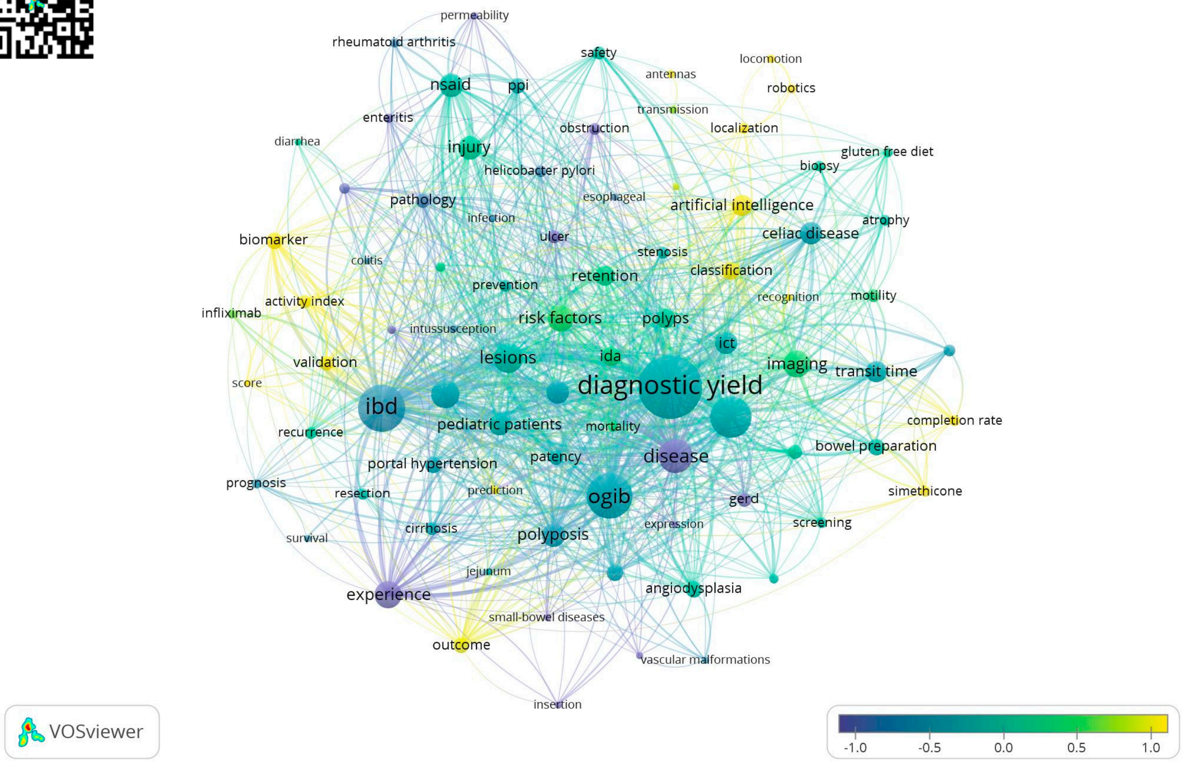

Supplement: Supplementary file 1 [file diagnostics-12-02238-s001.zip › diagnostics-1854020-supplementary.pdf]
